# Pyoderma caused by *Proteus mirabilis* in sheep

**DOI:** 10.1002/vms3.926

**Published:** 2022-09-01

**Authors:** Mostafa Abdollahi, Ashkan Jebelli Javan, Sara Shokrpoor, Mohammadhesam Beidokhtinezhad, Iraj Ashrafi Tamai

**Affiliations:** ^1^ Faculty of Veterinary Medicine Department of Internal Medicine University of Tehran Tehran Iran; ^2^ Faculty of Veterinary Medicine Department of Food Hygiene Semnan University Semnan Iran; ^3^ Faculty of Veterinary Medicine Department of Pathology University of Tehran Tehran Iran; ^4^ Faculty of Veterinary Medicine Department of Clinical Sciences Ferdowsi University of Mashhad Mashhad Iran; ^5^ Faculty of Veterinary Medicine Department of Microbiology and Immunology University of Tehran Tehran Iran

**Keywords:** pathology, *Proteus mirabilis*, pyoderma, sheep

## Abstract

Pyoderma is a purulent skin infection usually caused by bacteria and can be divided into primary and secondary categories based on histology. In the present study, an 18‐month‐old female mixed breed sheep was examined for pyoderma at the injection site of the enterotoxemia vaccine. After routine bacteriology and histopathology procedures, secondary pyoderma caused by *Proteus mirabilis* was diagnosed. The bacterium analysed using genome sequencing and new strain called AJJ 2021 was diagnosed. This is the first report of pyoderma caused by *Proteus mirabilis* in sheep.

## Graphical abstract

In the present study, an 18‐month‐old female mixed breed sheep with pyoderma (caused by new strain of *Proteus mirabilis*) was examined at the injection site of the enterotoxemia vaccine.

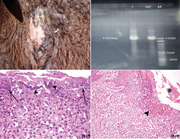

## 1 INTRODUCTION

Bacterial infections of the skin are usually purulent and are therefore commonly referred to as pyoderma. Pyoderma is classified into superficial and deep, depending on the level of tissue involvement (Denerolle et al., 1998). Pyoderma is further categorised as either primary or secondary. Primary pyoderma has no underlying cause in contrast to secondary pyoderma, in which the skin's health or integrity is compromised for other reasons, including an underlying disease process such as an endocrine or immunological disorder (Scott et al., 2012). Local disturbances of the skin's barrier integrity can also form a suitable ground for the occurrence of secondary pyoderma. These can include external parasite bites, rashes, fissures, other skin injuries or the entry of foreign objects such as plant thorns (Scott et al., 2011). This study describes the first report of secondary pyoderma caused by *Proteus mirabilis* in a sheep.

## 2 CASE HISTORY

**FIGURE 1:**
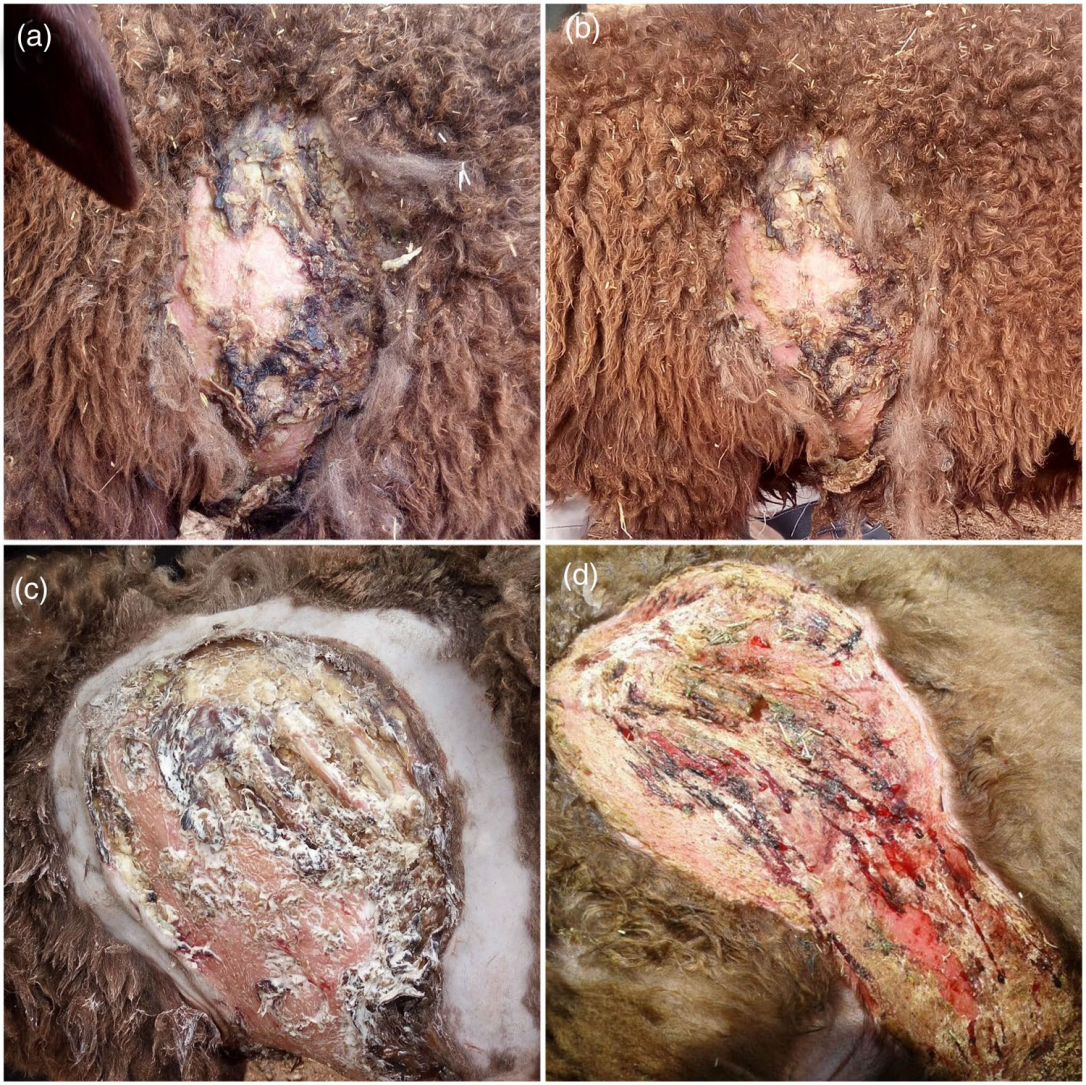
(a–d) Gross pathology findings of pyoderma caused by *Proteus mirabilis* in a sheep. (a, b) Necropurulent lesion of skin. (c) Extent of the lesion after shaving the wool of lesion site and applying the first topical treatment, including 1% povidone iodine solution, 1% silver sulphadiazine ointment, 1% phenytoin ointment. (d) Fourteen days after the start of treatment and collagen deposition at the lesion site

In February 2020, an 18‐month‐old female crossbred sheep was examined at Alian farm in Sorkheh city (Iran) for a skin lesion. This closed farm was in a desert area and had 170 breeding sheep. All animals were fed with hay (50%) and grain materials (50%) twice a day. Daily DM was 3%. The affected sheep was anorexic, but other animals had a normal appetite. There was no history of external parasites, itching and poor quality of the sheep external coating. In affected sheep, the skin lesion was located behind the scapula and was necropurulent in nature (Figure 1a and b). There was no previous treatment for this lesion. The lesion was painful to the touch. The history revealed that an enterotoxemia polyvalent vaccine (Razi Institute, Alborz, Iran) containing bacterial toxoids for *Clostridium perfringens* types b, c and d and *Clostridium septicum* was injected at the lesion site ten days before the examination. The vaccine was injected subcutaneously using a non‐sterile needle (the disposable needle had been used several times in the past and kept in an unclean box). This animal had been previously vaccinated with the same vaccine at 3 months of age. With the exception of lesion and anorexia, all vital signs were within normal limits and all other physical examination parameters where normal.

### 2.1 Bacterial culture

Without any prior preparation, bacteriological specimens consisted of two pre‐sterile pre‐labelled cotton swabs were rolled over the centre of the lesion and placed into Cary‐Blair transport medium and sent to the laboratory in a cool box containing frozen ice packs. The collected samples were inoculated on blood agar (Merck, Germany) and purified bacterial isolates streaked on differential (MacConkey agar, Oxoid) and selective (Proteeae isolation medium agar, Oxoid) media and incubated at 37°C for 24 h. All isolates were triply cloned to get new colonies for Gram staining, biochemical tests (TSI, Urea, Indole, Methyl red, Voges‐Proskauer, Simmon Citrate, Oxidase and Catalase) and PCR. The antibiotic sensitivity test of the isolate was performed.

### 2.2 Extraction and PCR

**FIGURE 2:**
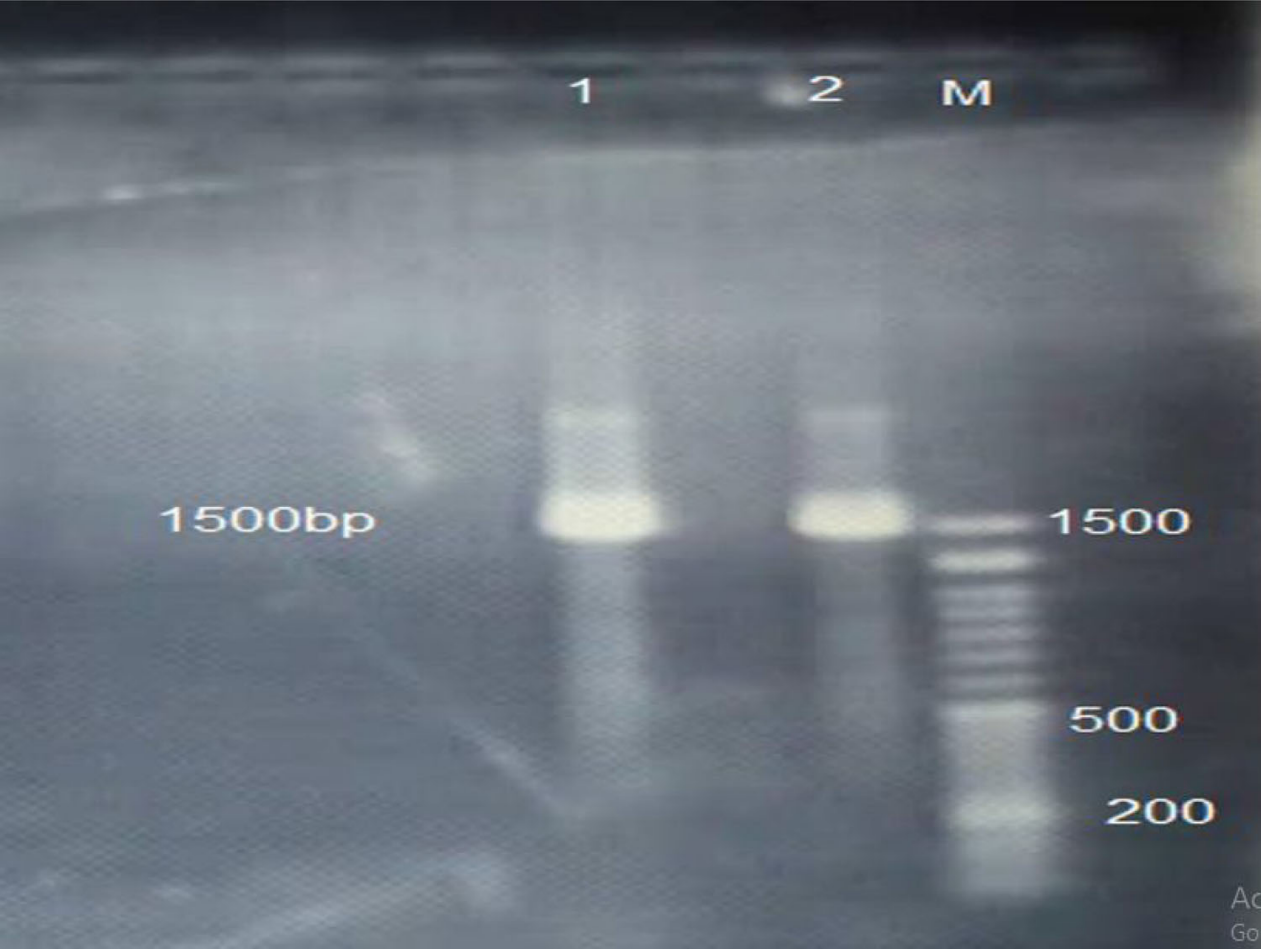
The band related to 16S rRNA gene band (1500 bp) in agarose gel electrophoresis in two samples 1 and 2

Isolated bacteria were cultured in TSB broth (Merck, Germany) and incubated at 37°C for 24 h. After that, 3 ml of TSB broth was centrifuged at 10,000 *g* for 10 min at 4°C. The pellet was washed once with saline solution. Finally, genomic DNA was extracted using a commercial DNA extraction kit for Gram‐negative bacteria according to the manufacturer's instruction (Sina clone, Iran). The *16S rRNA* gene was amplified using related primers (27F, 5'‐AGAGTTTGATCMTGGCTCAG‐3’, 1541R 5'‐AAGGAGGTGATCCAGCCGCA‐3’). PCR mixture was prepared using 12.5 µl of 2× Master Mix (Amplicon, Danmark), 0.2 pmol of the primer (100 pmol/µl), 1 µl of template DNA (100 ng) and 9.5 µl of distilled water in a final volume of 25 µl. The reaction was carried out as follows; an initial denaturation at 95°C for 3 min, then 34 cycles of 95°C for 30 min, 58°C for 30 min, 72°C for 90 s and a final extension at 72°C for 7 min. The amplification products (5 µl) were resolved by electrophoresis on 1.2% agarose gel for 1 h at 90 V. Afterwards, the agarose gel was stained with ethidium bromide and screened using UV‐illuminator. The amplified products were approximately 1500 bp (Figure 2) (Zhang et al., 2013). Sanger dideoxy sequencing method was used to obtain this sequence. The sequence derived from the isolate was analysed using the sequence analysis by Bioedit version 7 and BLAST search program (http://blast.ncbi.nlm.nih.gov/Blast.cgi), and this sequence was submitted to the Bankit. The accession number is MZ314326.

### 2.3 Histopathology

Three samples (0/5×0/5 cm) were taken from the lesion margin with disposable biopsy punches and placed in 10% neutral buffered formalin and sent to the histopathological laboratory. The samples routinely processed, dehydrated, embedded in paraffin wax, sectioned at 5 µm in thickness using a rotary microtome (RM2 145; Leica, Wetzlar, Germany) and stained with haematoxylin and eosin (H&E) for histopathological evaluation.

### 2.4 Treatment

The distance between the animal and the veterinary diagnostic laboratory was distance (>300 km), for this reason empirical treatment was initiated and prescribed while diagnostic results were pending. The wool around the lesion was sheared and cleaned using sterile gauze impregnated with 0.9% sodium chloride sterile solution. The lesion was debrided of necrotic debris using tissue forceps. Topical therapies applied  to improve wound healing and treat infection, included 1% povidone iodine (q12h, for 10 days, Rooyan Company, Iran), 1% silver sulphadiazine ointment (q12h, for 10 days, Najo Company, Iran), 1% phenytoin sodium ointment (q12h, for 10 days, Kish Medipharm Company, Iran) (Figure 1c). The was administered Pen‐Strep (containing 200 mg of procaine penicillin and 250 mg of dihydroestreptomycin sulphate per ml, 1 ml/25 kg, IM, q24h, for 3 days, Norbrook company, Iran) was initiated. Additionally, a single injection of AD3E (4 ml, SC, Rooyan Company, Iran) was administered at the visit.

### 2.5 Diagnostic results

**FIGURE 3:**
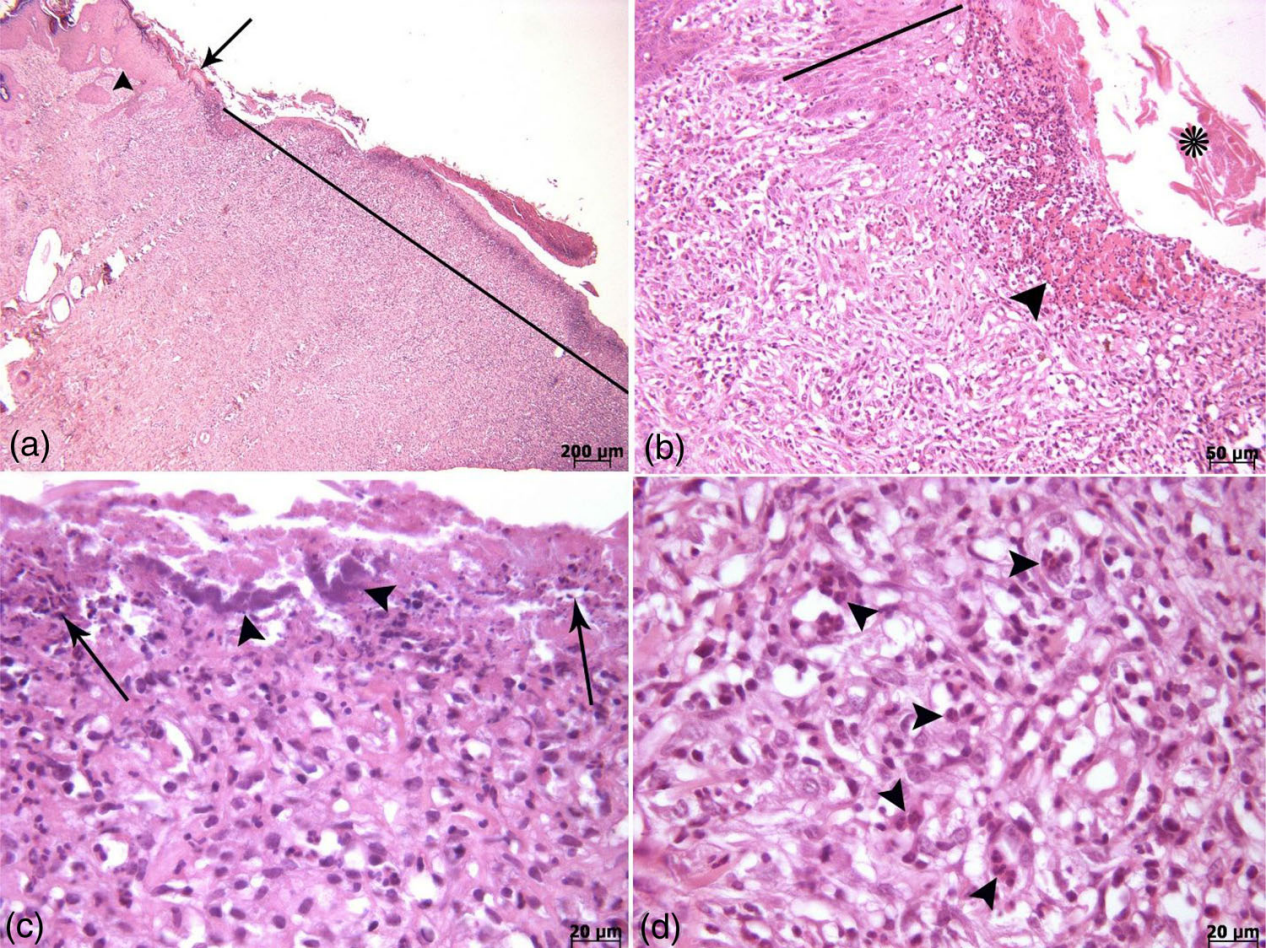
(a–d) Microscopical findings of pyoderma in a sheep. (a) Ulcer (black line), epidermal hyperplasia (acanthosis) (arrowhead), orthokeratotic and parakeratotic hyperkeratosis (arrow), ×40. (b) Acanthosis (black line), necrotic keratinocytes (arrowhead), cellular exudate and sloughed epidermal cells (*), ×200. (c) Basophilic bacterial colonies (arrowheads) and debris cell (arrows), ×400. (d) Neutrophils (arrowheads) in the dermal layer, ×400, H&E

Histopathologically, moderate epidermal hyperplasia, orthokeratotic and parakeratotic hyperkeratosis were observed. There was severe ulceration, fluid debris, cellular exudate, degenerate neutrophils, necrotic keratinocytes, and basophilic bacterial colonies were also present (Figure 3a–c). In the dermal layer, interstitial neutrophilic to mixed mononuclear inflammation and destruction of adnexal units (follicles and sebaceous glands) were seen (Figure 3d). These findings were consistent with a diagnosis of deep pyoderma.

The underlying bacterial cause of the pyoderma was determined to be *Proteus mirabilis*. *Proteus mirabilis* was identified through differential and selective media, Gram‐staining and biochemical tests, as shown in Table 1 (Zafar et al., 2019). Antibiotic susceptibility testing showed that the isolate was sensitive to cefixime, florfenicol, streptomycin, gentamicin, sulphadiazine, neomycin and cefepime; intermediately sensitive to ciprofloxacin, enrofloxacin and colistin; and resistant to tetracycline, minocycline, ceftazidime, penicillin, erythromycin and azithromycin antibiotics. Using genome sequencing a new *Proteus mirabilis* strain was characterised and in termed AJJ 2021.

**TABLE 1:** Biochemical Characterization of *Proteus mirabilis* Isolated from sheep pyoderma

	In MacConkey agar	Non‐lactose fermenting, large, circular smooth colonies
Cultural	In proteeae isolation medium (PIM) agar	Dark brown colonies with a zone of clearing in the medium
	Gram staining	–
	Shape	Rods
	Size	Length: 1–3 µm/width: 0.4–0.8 µm
	Motility	+
	Indole	–
	TSI	Alk/A, H2S+
	Urea	+
	Citrate utilisation	+/–
Biochemical	MR	+
	VP	–
	Urease	+
	Catalase	+
	Oxidase	–
	Glucose	+
	Trehalose	+
	Lactose	–
Sugar fermentation	Sucrose	–
	Mannitol	–
	Dulcitol	–
	Sorbitol	–
	Xylose	+
	Inositol	–

*Note*: Positive (+), negative (–) and variable (+/–).

### 2.6 Outcome and follow‐up

The results from the culture and antibiotic susceptibility testing indicated that the AJJ 2021 strain of *Proteus mirabilis* was sensitive to dihydroestreptomycin sulphate and silver sulphadiazine. A positive response to treatment was observed and 2 weeks after the initial visit the lesion had the wound contracting (Figure 1d). During the 1‐year follow‐up via the phone, the wound had completely healed, leaving a persistent scar. The sheep was apparently health and gone through a successful gestation period.

## 3 DISCUSSION

The present report found that the referred sheep suffered from pyoderma caused by *Proteus mirabilis*. The Gram‐negative bacterium *Proteus mirabilis* is a transient organism that cannot reproduce on healthy skin and is considered a secondary invader in causing pyoderma (Armbruster et al., 2018; Jeong & Oh, 2011). Some predisposing factors, such as allergy, seborrhoea, immune deficiency, injection of vaccine and other causes of follicular inflammation or dysfunction often play a primary role (Hargis & Myers, 2017). During the authors’ search of scientific sources, no other report was found of the pyoderma caused by *Proteus mirabilis* in sheep. *Staphylococcus* and *Dermatophilus congolensis* are more common causes of pyoderma in sheep (Constable et al., 2016). Few published reports of pyoderma caused by Proteus species are described in animals and humans. To date, pyoderma caused by Proteus species has been identified in humans and dogs. Jae Lee et al. (2015) reported the incidence of *Gangrenosum ecthyma* (GE) caused by *Proteus vulgaris* and *Candida albicans* in a 57‐year‐old woman with Castleman disease. The most common etiological agent of canine pyoderma is *Staphylococcus pseudintermedius*. The patient had suffered from recurrent pyoderma for years and underwent numerous treatments based on clinical findings without paraclinical examination. The cytology of the lesion made pyoderma suspicious. Bacterial culture, accurate identification of pathogens (*Proteus vulgaris* and *Staphylococcus pseudintermedius*) and treatment based on laboratory findings led to complete recovery was achieved with prolonged administration of fluorquinolones (Jeong & Oh, 2011).

In this report, the observed pyoderma was classified as secondary and deep pyoderma. Subcutaneous injection of an enterotoxemia vaccine can cause a local swelling due to inflammation secondary to a foreign substance (Armbruster et al., 2018). Indeed, minor reactions to inactivated vaccines may result from the irritant nature of some adjuvants, such as aluminium (Chung, 2014) which is the adjuvant in the vaccine administered to this sheep before developing the lesion. Other causes for localised vaccine reactions include the immunising antigens, conjugating agents, preservatives, stabilisers, antimicrobial agents, and culture media used to prepare the vaccine (Chung, 2014). Further, according to the manufacturer of the specific vaccine used to inoculate this sheep, the vaccine may cause local allergic reactions. Changes in the skin's barrier integrity due to a local response to the vaccine at the injection site could have predisposed this sheep to bacterial pyoderma. According to the history of this patient, a non‐sterile needle was used to administer the vaccine, which could have caused contamination leading to pyoderma is also a possibility. In the case of this sheep, it is difficult to determine if the underlying cause was the non‐sterile needle, the vaccine or a combination of both.

Based on the results of the antibiotic sensitivity test of the isolate, among the antibiotics used in this study, two (dihydroestreptomycin sulphate and silver sulphadiazine) were effective antibiotics and one (procaine penicillin) was ineffective antibiotics against isolated *Proteus mirabilis*.

## 4 CONCLUSION

This study showed that *Proteus mirabilis* can cause pyoderma in sheep and should be considered as a possible cause for pyoderma. The isolated *Proteus mirabilis* was sequenced for the 16S rRNA genome and registered as a new strain in the NCBI gene bank with code MZ314326 and AJJ 2021.

## AUTHOR CONTRIBUTIONS

Mostafa Abdollahi: Conception, performing the experiment, writing the article. Ashkan Jebelli Javan: Conception and design, collecting the data, writing and revising the article. Sara Shokrpoor: Assistance in laboratory work. Mohammadhesam Beidokhtinezhad: Assistance in performing the experiment. Iraj Ashrafi Tamai: Assistance in laboratory work.

## CONFLICT OF INTEREST

The authors declare that there is no conflict of interest.

## FUNDING INFORMATION

There is no source of fund to declare.

### PEER REVIEW

The peer review history for this article is available at https://publons.com/publon/10.1002/vms3.926.

## ETHICAL CONSIDERATION

After sampling, the code of ethics in the research was taken from research committee of Alian Sheep Breeding Company (ethics code: 0061891274).

## ETHICAL APPROVAL

The authors confirm that the ethical policies of the journal, as noted on the journal's author guidelines page, have been adhered to and the appropriate ethical review committee approval has been received.

## Data Availability

The data that supports the findings of this study are available within the manuscript and also are available from the corresponding author upon reasonable request.

## References

[vms3926-bib-0001] Armbruster, C. E. , Mobley, H. L. T. , & Pearson, M. M. (2018). Pathogenesis of *Proteus mirabilis* Infection. EcoSal Plus Journal, 8, 1–123. 10.1128/ecosalplus.esp-0009-2017 PMC5880328 29424333

[vms3926-bib-0002] Chung, E. H. (2014). Vaccine allergies. Clinical and Experimental Vaccine Research, 3, 50–57. 24427763 10.7774/cevr.2014.3.1.50 PMC3890451

[vms3926-bib-0003] Constable, P. D. , Hinchcliff, K. W. , Done, S. H. , & Grunberg, W. (2016). Diseases of the epidermis and dermis in veterinary medicine (11th ed., pp. 1947). Philadelphia: Saunders Elsevier.

[vms3926-bib-0004] Denerolle, P. , Bourdoiseau, G. , Magnol, J. P. , Ulpat, C. , & Chabanne, L. (1998). German shepherd dog pyoderma: A prospective study of 23 cases. Veterinary Dermatology, 9, 243–248.

[vms3926-bib-0005] Hargis, A. M. , & Myers, S. (2017) The integument in pathologic basis of veterinary disease (6th ed., pp. 1071). Missouri, USA: Mosby.

[vms3926-bib-0006] Jae Lee, Y. , Jung, I. O. , & Oh, D. Y. (2015). A rare case of ecthyma gangrenosum caused by *Proteus vulgaris* and *Candida albicans* in a patient with Castleman disease. Archives of Plastic Surgery, 42, 805–807. 26618137 10.5999/aps.2015.42.6.805 PMC4660003

[vms3926-bib-0007] Jeong, H. H. , & Oh, T. H. (2011). Recurrent superficial pyoderma caused by mixed infection of *Proteus mirabilis* and *Staphylococcus pseudointermedius* in a Yorkshire terrier dog. Journal of Veterinary Clinics, 28, 538–541.

[vms3926-bib-0008] Scott, D. W. , Miller, W. H. , & Erb, H. N. (2012). Feline dermatology at Cornell University: 1407 cases (1988‐2003). Journal of Feline Medicine and Surgery, 15, 307–316. 23186638 10.1177/1098612X12468922 PMC10816771

[vms3926-bib-0009] Scott, D. W. , Vogel, J. W. , Fleis, R. I. , Miller, W. H. Jr. , & Smith, M. C. (2011). Skin diseases in the alpaca *(Vicugna pacos)*: A literature review and retrospective analysis of 68 cases (Cornell University 1997‐2006). Veterinary Dermatology, 22, 2–16. 20825592 10.1111/j.1365-3164.2010.00918.x

[vms3926-bib-0010] Zafar, U. , Taj, M. K. , Nawaz, I. , Zafar, A. , & Taj, I. (2019). Characterization of *Proteus mirabilis* isolated from patient wounds at Bolan Medical Complex Hospital, Quetta. Jundishapur Journal of Microbiology, 12, e87963.

[vms3926-bib-0011] Zhang, W. , Niu, Z. , Yin, K. , Liu, P. , & Chen, L. (2013). Quick identification and quantification of *Proteus mirabilis* by polymerase chain reaction (PCR) assays. Annals of Microbiology, 63, 683–689.

